# Atypical drug-induced hypersensitivity syndrome with multiple organ failure rescued by combined acute blood purification therapy: a case report

**DOI:** 10.1186/s12245-023-00511-2

**Published:** 2023-05-09

**Authors:** Hideaki Oiwa, Shozo Yoshida, Hideshi Okada, Masahiro Yasunishi, Ryo Kamidani, Kodai Suzuki, Takahito Miyake, Tomoaki Doi, Takayoshi Shimohata, Shinji Ogura

**Affiliations:** 1 Department of Emergency and Disaster Medicine, Gifu University Graduate School of Medicine, Gifu, 501-1194 Japan; 2 Abuse Prevention Center, Gifu University Graduate School of Medicine, Gifu, Japan; 3 Department of Neurology, Gifu University Graduate School of Medicine, Gifu, Japan

**Keywords:** Drug-induced hypersensitivity syndrome, Acute blood purification therapy, Acute kidney injury, Plasma exchange, Multi-organ failure

## Abstract

**Background:**

Drug-induced hypersensitivity syndrome (DIHS), including Stevens-Johnson syndrome (SJS), is a severe rash that often develops 2–6 weeks after the intake of the causative drug; however, its diagnosis is sometimes difficult. This article describes a case in which a patient with DIHS-induced multiple organ failure was successfully treated with blood purification therapy.

**Case presentation:**

A male patient in his 60s was admitted to our hospital with autoimmune encephalitis. The patient was treated with steroid pulse therapy, acyclovir, levetiracetam, and phenytoin. From the 25th day, he presented with fever (≥ 38 °C) as well as miliary-sized erythema on the extremities and trunk, followed by erosions. DIHS and SJS were suspected; accordingly, levetiracetam, phenytoin, and acyclovir were discontinued. On the 30th day, his condition further deteriorated, and he was admitted to the intensive care unit for ventilatory management. The next day, he developed multi-organ failure and was started on hemodiafiltration (HDF) for acute kidney injury. Although he presented with hepatic dysfunction and the appearance of atypical lymphocytes, he did not meet the diagnostic criteria for DIHS or SJS/toxic epidermal necrolysis. Therefore, he was diagnosed with multi-organ failure caused by severe drug eruption and underwent a 3-day treatment with plasma exchange (PE) in addition to HDF. Accordingly, the patient was diagnosed with atypical DIHS. After being started on blood purification therapy, the skin rash began to disappear; moreover, the organ damage improved, with a gradual increase in urine output. Eventually, the patient was weaned off the ventilator and transferred to the hospital on the 101st day.

**Conclusions:**

HDF + PE could effectively treat multi-organ failure caused by atypical DIHS, which is difficult to diagnose.

## Background

Drug-induced hypersensitivity syndrome (DIHS) is a severe drug rash similar to Stevens-Johnson syndrome (SJS) and toxic epidermal necrolysis (TEN) [1, 2]. 

It has a high mortality rate (10–20%) and is caused by a relatively limited number of drugs, including carbamazepine, phenytoin, phenobarbital, zonisamide, allopurinol, salazosulfapyridine, diaphenyl sulfone, mexiletine, and minocycline [3]. There remains no established treatment for DIHS; however, its symptoms can be addressed by discontinuation of the suspected drugs, systemic steroids, steroid pulse therapy in severe cases, and supportive care such as blood purification for organ damage [4].

The symptoms of DIHS include fever, sore throat, general malaise, and loss of appetite. However, unlike ordinary drug eruptions, a prompt definitive diagnosis may be difficult since symptoms may develop at least 2 weeks after treatment initiation [3].

## Case presentation

**Fig. 1:**
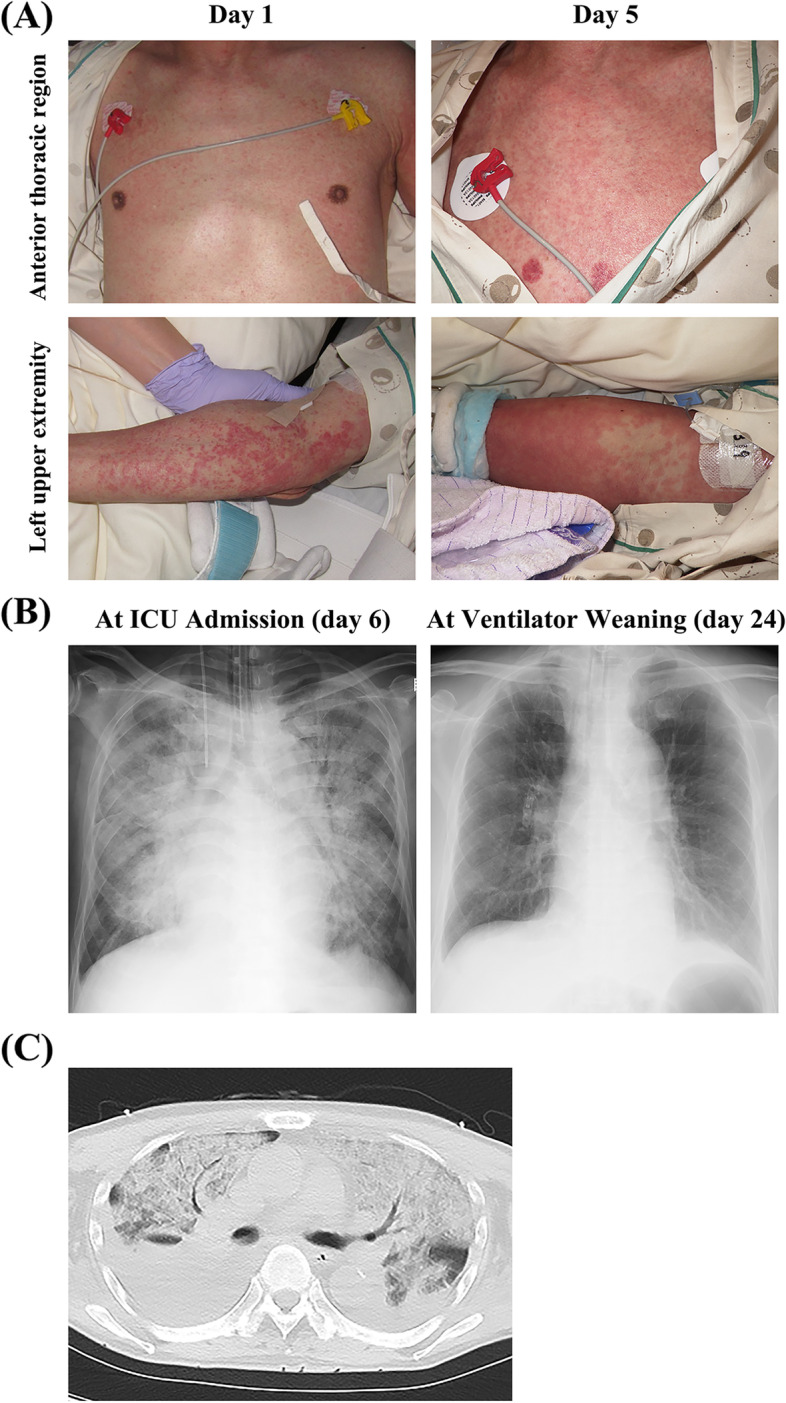
Skin findings on day 1 and day 5 and chest radiograph and CT. Lesions in **A** the anterior thoracic region and upper left extremity. Miliary-sized erythema was observed on the extremities and trunk on day 1 (**A**). On day 5, there was expansion of the skin rash. **B** Chest radiography upon ICU admission (day 6, left panel) and ventilator weaning (day 24, right panel). The decreased permeability of the entire lung field, which could have been caused by the infiltrated fluid observed at ICU admission, improved at the time of weaning from ventilation. **C** Chest computed tomography (CT) at ICU admission. Frosted shadows in all lung fields and a large pleural effusion were observed

An Asian male patient in his 60s with autoimmune encephalitis was admitted to the neurology department. The patient had a history of hypertension and no history of diabetes. There was no family history. He did not have a smoking history. He was treated with steroid pulse therapy, acyclovir, levetiracetam, phenytoin, tazobactam/piperacillin, ampicillin/sulbactam, and vancomycin (VCM) for aspiration pneumonia and urinary tract infections that subsequently occurred. Although his consciousness disorder and general condition improved on the 37th day of admission (day 1), he developed a fever (≥ 38 °C) and scattered miliary-sized erythema on the extremities and trunk, with some showing fusion (Fig. 1A, left panels). There was no erythema on the face; additionally, there were no abnormalities in the ocular or oral mucosa.

Since drug eruptions, including DIHS and SJS, were suspected, levetiracetam and phenytoin were discontinued, and topical steroids were started. On day 3, the suspected drug VCM was changed to linezolid. However, on day 5, his skin rash showed expansion (Fig. 1A, right panels), and he was started on steroid pulse therapy (methylprednisolone [mPSL] 1000 mg/day for 3 days). Moreover, his respiratory condition rapidly worsened, and he was started on noninvasive-positive pressure ventilation (NPPV). Drug-induced lymphocyte stimulation tests (DLST) were performed on levetiracetam and phenytoin on day 5.

On day 6, the NPPV could no longer keep the patient oxygenated. Upon admission to the intensive care unit (ICU), his Glasgow Coma Scale score was 14 (eye, 4; verbal, 4; motor, 6). Physical examination revealed a body temperature of 38.5 °C, tachypnea (respiratory rate: 20 breaths/min), a heart rate of 103 bpm, and a blood pressure of 129/57 mmHg. On auscultation, there was a decrease in breath sounds and fine crackles in all lung fields; however, there was no heart murmur.

Laboratory tests upon admission to the ICU revealed an elevated inflammatory response (C-reactive protein: 16.7 mg/dL), increased levels of liver enzymes (aspartate transaminase, 48 IU/L; alanine aminotransferase, 86 IU/L), and decreased renal function (blood urea nitrogen, 30.6 mg/dL; creatinine, 1.17 mg/dL) (Table 1). Arterial blood gas analysis using NPPV revealed a decrease in the PaO_2_/F_I_O_2_ ratio (62.9 at F_I_O_2_ 1.0). Chest radiography showed decreased permeability in both lung fields (Fig. 1B); furthermore, chest computed tomography revealed frosted shadows in all lung fields and extensive pleural effusion (Fig. 1C). He was placed on a ventilator due to multi-organ failure caused by severe drug eruption. At this point, the patient did not meet the diagnostic criteria for DIHS or SJS/TEN (Table 2); therefore, treatment was initiated for multi-organ failure due to severe drug eruption.

**Table 1:** Laboratory findings at the time of ICU admission

** < CBC > **			** < Biochemistry > **		
White blood cells	16,730	/uL	Total protein	5.4	g/dL
Neutrophil	96	%	Albumin	2.2	g/dL
Monocyte	2.1	%	Creatinine kinase	47	g/dL
Lymphocyte	0.5	%	Aspartate transaminase	48	IU/L
Eosinophil	0	%	Alanine transaminase	86	IU/L
Basophil	0	%	Alkaline phosphatase	427	IU/L
Atypical lymphocyte	1	%	*γ*-Glutamyl transpeptidase	252	IU/L
			Creatinine	1.17	mg/dL
Red blood cells	5.36 × 10^6^	/uL	Blood urea nitrogen	30.6	mg/dL
Hemoglobin	9.1	dL	Total bilirubin	0.5	mg/dL
Hematocrit	26.5	%	Sodium	133	mEq/L
Platelet	13.7 × 10^4^	uL	Potassium	3.8	mEq/L
** < Coagulation status > **			Chlorine	97	mEq/L
APTT	48.7	s	Calcium	7.2	mg/dL
PT-INR	1.06		Phosphorus	4.0	mg/dL
Fibrinogen	228	mg/dL	Magnesium	1.9	mg/dL
D-dimer	4.7	ug/mL	C-reactive protein	0.55	mg/dL
FDP	10.4	ug/mL	Blood glucose	223	mg/dL
Antithrombin III	57	%			
** < Venous blood gas > **					
F_I_O_2_	1.0				
pH	7.38		APACHE II score	16	
PaCO_2_	46.7	mmHg	SOFA score	9	
PaO_2_	62.9	mmHg	DIC score^a^	1	
HCO_3_^−^	27.2	mmol/L			
Base excess	2.3				
Lactate	24	mg/dL			

^a^Established by the Japanese Association for Acute Medicine

**Table 2:** Diagnostic criteria for DIHS and SJS/TEN

**The diagnostic criteria for DIHS**	**Day 6**	**Day 14**
1) Delayed erythema onset following administration of a limited number of medications, which rapidly enlarge and often progress to erythroderma	✓	✓
2) Persistence for > 2 weeks after discontinuation of the causative agent		✓
3) Fever > 38 °C	✓	✓
4) Hepatic dysfunction	✓	✓
5) One or more of the following:		
a)Leukocyte proliferation > 11,000/mm^3^ b) Atypical lymphocytes (> 5%) or eosinophils (> 1500/mm^3^)	✓	✓
6) Lymphadenopathy		
7) Reactivation of HHV-6		
Typical DIHS meets all, and atypical DIHS meets (1)–(5)		
**The diagnostic criteria for SJS/TEN**	**Day 6**	**Day 14**
1) Extensive mucosal involvement of the cutaneous-mucosal transition zone		
2) Erosions or blisters covering < 10% of the body surface area (SJS)/ > 10% (TEN)	✓	✓
3) Fever up	✓	✓
4) Necrotic changes in the epidermis on pathology		
5) Denial of severe forms of erythema multiforme	✓	✓

SJS/TEN is diagnosed when all of these five criteria are met

**Fig. 2:**
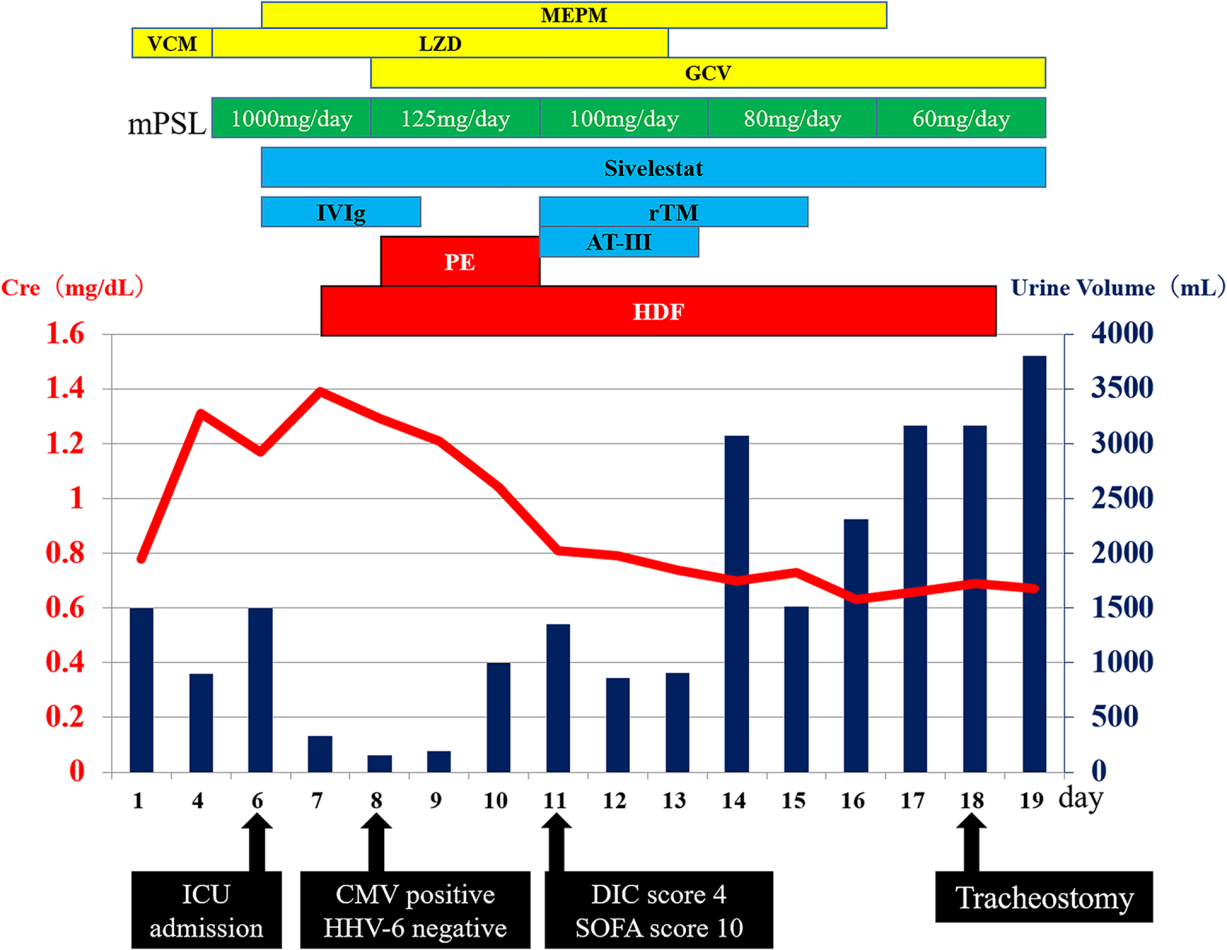
Clinical course. MEPM, meropenem; VCM, vancomycin; LZD, linezolid; GCV, ganciclovir; mPSL, methylprednisolone; IVIg, intravenous immunoglobulin; rTM, recombinant thrombomodulin; AT III, antithrombin III; PE, plasma exchange; HDF, hemodiafiltration; CMV, *Cytomegalovirus*; HHV-6, human herpesvirus 6; Cre, creatinine

After admission to the ICU, the patient underwent 3-day treatment using intravenous immunoglobulin 5.0 g/day, meropenem, and sivelestat for severe drug eruption, severe pneumonia, or acute respiratory distress syndrome (Fig. 2). However, on day 7, his urine output rapidly declined; furthermore, he was diagnosed with KDIGO stage 3 acute kidney injury and was started on hemodiafiltration (HDF) using a polysulfone high-performance membrane (FDZ-21, Nikkiso, Tokyo, Japan) for 6 h daily. The blood, dialysate, and filtrate flow rates were maintained at 150–180 ml/min, 500 ml/min, and 25 ml/kg/daily, respectively. Sublood-BS (Fuso Pharmaceutical, Osaka, Japan) was used as dialysate. On day 8, plasma exchange (PE) was performed for 3 days to ensure an effect on DIHS given the suspected multi-organ failure and SJS. PE was performed using a membrane plasma separator (OP-08, Asahi Kasei Medical, Tokyo, Japan) for 3 h daily with HDF. On the same day, steroid pulse therapy was terminated; accordingly, the mPSL dose was reduced to 125 mg/day and then tapered off. On day 8, the patient tested negative for human herpesvirus 6 (HHV-6), which was a diagnostic criterion; however, the patient tested positive for *Cytomegalovirus* (CMV) and was started on ganciclovir. However, by day 11, he had an acute disseminated intravascular coagulation score of 4; therefore, recombinant human thrombomodulin was administered for 5 days, and recombinant antithrombin III formulation was administered for 3 days. On day 14, the patient met the diagnostic criteria for atypical DIHS (Table 2). Subsequently, the patient’s general condition improved, with HDF being terminated on day 18. A tracheostomy was performed on the same day. The ventilator was removed on day 24, and the patient was discharged from the ICU on day 28 and was then transferred to the hospital on day 68 after symptom improvement.

## Discussion and conclusions

Severe cutaneous adverse reactions to pharmaceuticals include various conditions, including DIHS and SJS/TEN [2]. There are seven and five diagnostic criteria for DIHS and SJS/TEN, respectively (Table 2) [1, 4]; however, both DIHS and SJS/TEN result in severe systemic symptoms; moreover, there may be overlapping cases [5], which impedes diagnosis in some cases. Furthermore, the difficulty in the diagnosis of DIHS could be attributed to rash, fever, and liver lesions usually disappearing after discontinuation of the suspected drug [3]. Since several of these diagnostic criteria are time-consuming to determine, the differential diagnosis of DIHS and SJS/TEN was difficult for our patient.

This patient was finally diagnosed with atypical DIHS. Although DLST can become positive after 1 month in patients with DIHS, none of the drugs in our case showed DLST positivity. Accordingly, the causative agent remained unknown.

In addition to HHV-6, reactivation of CMV, Epstein-Barr virus, herpes simplex virus, and varicella-zoster virus has been reported in patients with DIHS [6, 7]. In our patient’s case, CMV reactivation was observed, but it was not known whether this was due to DIHS or steroid therapy, a known risk factor for CMV reactivation. In addition, the patient did not show symptoms of active CMV disease. Therefore, although treatment with ganciclovir was ongoing, specific testing such as PCR for CMV and HHV six in bronchoalveolar lavage was not performed.

Since our patient showed multi-organ failure, HDF and PE were both administered to allow an effect on either DIHS or SJS/TEN. After PE therapy, the patient’s general condition and skin condition improved. Therefore, PE should be considered for severe drug eruptions that can cause multi-organ failure.

We encountered a case of atypical DIHS with multiple organ failure, which was resolved by acute blood purification therapy. Diagnosis of severe drug eruptions may be difficult during the early disease stages; accordingly, the treatment protocol should consider each of the probable diseases.

## Data Availability

The datasets used and/or analyzed during the current study are available from the corresponding author on reasonable request.

## Abbreviations

CMV: *Cytomegalovirus*
DIHS: Drug-induced hypersensitivity syndrome
DLST: Drug-induced lymphocyte stimulation test
HDF: Hemodiafiltration
HHV-6: Human herpesvirus 6
mPSL: Methylprednisolone
NPPV: Noninvasive positive pressure ventilation
PE: Plasma exchange
SJS: Stevens-Johnson syndrome
TEN: Toxic epidermal necrolysis
VCM: Vancomycin
